# Effects of Elobixibat on Constipation and Lipid Metabolism in Patients With Moderate to End-Stage Chronic Kidney Disease

**DOI:** 10.3389/fmed.2021.780127

**Published:** 2022-01-17

**Authors:** Momoko Matsuyama, Keiji Hirai, Hiroaki Nonaka, Moeka Ueda, Junki Morino, Shohei Kaneko, Saori Minato, Yuko Mutsuyoshi, Katsunori Yanai, Hiroki Ishii, Taisuke Kitano, Akinori Aomatsu, Haruhisa Miyazawa, Kiyonori Ito, Yuichiro Ueda, Susumu Ookawara, Yoshiyuki Morishita

**Affiliations:** Division of Nephrology, First Department of Integrated Medicine, Saitama Medical Center, Jichi Medical University, Saitama, Japan

**Keywords:** elobixibat, constipation, lipid metabolism, cholesterol, chronic kidney disease, dialysis

## Abstract

**Objective:**

The aim of this study was to investigate the effects of elobixibat on constipation and lipid metabolism; and determine the factors associated with the effect of elobixibat on constipation in patients with moderate to end-stage chronic kidney disease (CKD).

**Methods:**

Stool frequency and serum lipid parameters were retrospectively analyzed before and after 4 weeks of elobixibat administration in 42 patients (CKD stage G3, 6; stage G4, 9; stage G5, 9; stage G5D, 18). Relationships between the change in stool frequency after initiation of elobixibat and various clinical parameters were analyzed by using linear regression analysis.

**Results:**

Elobixibat increased stool frequency from 0.5 ± 0.4 per day to 1.1 ± 0.6 per day (*p* < 0.001) regardless of whether patients were undergoing dialysis, on concomitant laxatives, or were administered elobixibat before or after breakfast. Elobixibat reduced low-density lipoprotein cholesterol concentration (from 90.9 ± 37.2 mg/dL to 77.5 ± 34.8 mg/dL, *p* < 0.05) and increased high-density lipoprotein cholesterol concentration (from 44.9 ± 14.3 mg/dL to 57.0 ± 25.8 mg/dL, *p* < 0.05), but did not change triglyceride concentration. Adverse effects were observed in two patients (nausea and diarrhea). Only phosphate concentration was correlated with the change in stool frequency after initiation of elobixibat (standard coefficient = 0.321, *p* = 0.043).

**Conclusions:**

Elobixibat improved constipation and lipid metabolism in patients with moderate to end-stage CKD, without serious adverse events.

## Introduction

Chronic constipation is a common complication in patients with chronic kidney disease (CKD) and is associated with progression of CKD, development of cardiovascular disease, poor quality of life, and frailty and sarcopenia (1–4). Therefore, optimal treatment of chronic constipation is important for preserving renal function, preventing development of cardiovascular events, improving quality of life, and mitigating progression of frailty and sarcopenia.

In Japan, magnesium oxide and anthraquinone stimulant laxatives have been widely used for treatment of chronic constipation (5). However, magnesium oxide laxatives are not recommended in patients with renal impairment because of increased risk of hypermagnesemia resulting from reduced urinary excretion of magnesium (6). Anthraquinone stimulant laxatives become less effective with long-term use and can cause melanosis coli, a dark brown-black pigmentation of the colonic mucosa (7).

Elobixibat is a novel laxative that inhibits bile acid transporters expressed in epithelial cells of the terminal ileum and increases the amount of bile acid flowing into the colon lumen (8). It increases water secretion into the large intestine lumen and promotes intestinal motility. Elobixibat does not induce drug resistance, unlike stimulating laxatives, and can be used safely in patients with renal impairment (8). A recent randomized study showed that elobixibat increased the frequency of spontaneous bowel movements in patients with functional constipation (9). Another study reported that elobixibat reduced low-density lipoprotein (LDL)-cholesterol concentration in Japanese patients with chronic constipation (10). However, few studies have investigated the effects of elobixibat on constipation and lipid metabolism in patients with renal impairment. Furthermore, the factors associated with the effect of elobixibat on constipation have yet to be determined. Therefore, in the present study, we investigated the factors associated with the effect of elobixibat on constipation, as well as the effects of elobixibat on constipation and lipid metabolism in patients with moderate to end-stage CKD.

## Materials and Methods

### Ethical Approval

This study was approved by the ethics committee of Saitama Medical Center, Jichi Medical University (RIN 15-33), and was conducted according to the principles of the Declaration of Helsinki. Informed consent was not required because of the retrospective nature of the study. Information regarding this study was displayed on notice boards in the patient waiting rooms of our institution to inform patients of their right to opt out.

### Patients

We analyzed data from patients who were treated at Saitama Medical Center, Jichi Medical University, between 2018 and 2020. The inclusion criteria were as follows: (i) age over 20 years, (ii) diagnosed with chronic functional constipation based on Rome IV criteria (11), (iii) estimated glomerular filtration rate ≤ 60 mL/min/1.73 m^2^ (CKD stage G3–G5D), and (iv) taking elobixibat for 4 weeks or longer. The exclusion criterion was as follows: (i) patients with malignancy.

### Study Design

**Figure 1:**
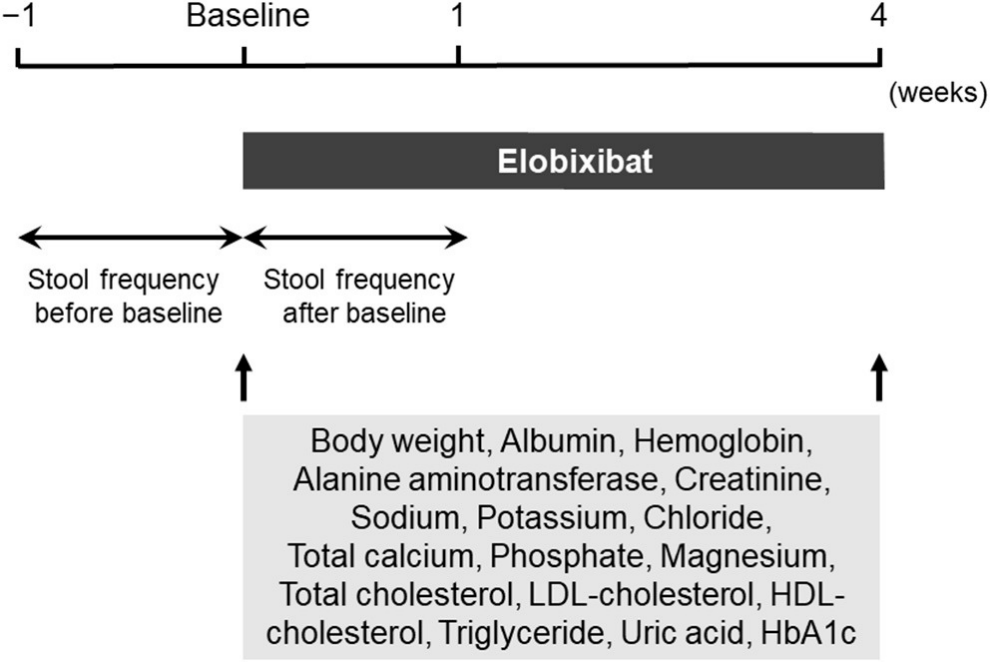
Study design diagram. HbA1c, glycosylated hemoglobin; HDL, high-density lipoprotein; LDL, low-density lipoprotein.

This was a retrospective study involving 42 patients. The study design is illustrated in Figure 1. Demographic parameters and clinical data of patients were retrospectively obtained from medical records. Elobixibat was administered orally once per day, before or after breakfast. Elobixibat was started at a dose of 10 mg/day, and increased to 15 mg/day if not sufficiently effective, or decreased to 5 mg/day if too effective. A recent phase III clinical study reported that spontaneous bowel movements were observed within 24 h after first administration of 10 mg/day of elobixibat in ~90% of the patients (9). Therefore, elobixibat was increased to 15 mg/day when there was no bowel movement 24 h after initiation of 10 mg/day of elobixibat. In contrast, elobixibat was decreased to 5 mg/day when diarrhea, defined as three or more bowel movements (12), was observed 24 h after initiation of 10 mg/day of elobixibat. Stool frequency was assessed 1 week before and after initiation of elobixibat. Lipid parameters, including circulating concentrations of total cholesterol, LDL-cholesterol, high-density lipoprotein (HDL)-cholesterol, and triglyceride were evaluated before and after 4 weeks of elobixibat administration. The factors that were associated with the change in stool frequency after initiation of elobixibat were identified using linear regression analysis.

### Assessment of Stool Frequency

Stool frequency was defined as the number of spontaneous bowel movements per day. The average number of spontaneous bowel movements per day was measured for 1 week before and after initiation of elobixibat administration in each patient. The change in stool frequency after initiation of elobixibat was calculated by subtracting stool frequency before from after initiation of elobixibat.

### Laboratory Methods

Blood and urinary parameters were measured at the Department of Clinical Laboratory, Saitama Medical Center. Serum glycosylated hemoglobin (HbA1c) concentrations are presented as National Glycohemoglobin Standardization Program values (%). Diabetes mellitus was defined as taking oral hypoglycemic agents and/or insulin therapy, or serum HbA1c levels ≥ 6.5%.

### Statistical Analysis

Statistical analyses were performed using JMP v11 (SAS Institute, Cary, North Carolina, USA). Data are presented as mean ± standard deviation for continuous variables, and as number and percentage for categorical variables. Comparisons of data before and after administration of elobixibat were performed using the paired *t*-test. Relationships between the change in stool frequency after initiation of elobixibat and clinical parameters were examined using linear regression analysis. *p* < 0.05 was considered statistically significant.

## Results

### Patient Features

Baseline patient features and medications are shown in Table 1. A total of 42 patients (30 men and 12 women; mean age: 72.5 ± 13.6 years; body mass index: 23.0 ± 3.7 kg/m^2^) were analyzed. Mean serum creatinine at baseline was 5.2 ± 3.6 mg/dL, and CKD stages were as follows: stage G3, 6 (14.3%); stage G4, 9 (21.4%); stage G5, 9 (21.4%); and stage G5D, 18 (42.9%). Of 18 patients with CKD stage G5D, 15 were undergoing hemodialysis and three were undergoing peritoneal dialysis. Eighteen (42.9%) patients had diabetes mellitus (Table 1). The proportions of medications used among patients were as follows: dipeptidyl peptidase-4 inhibitor, 21.4%; glucagon-like peptide-1 receptor agonist, 0.0%; insulin, 14.3%; metformin, 0.0%; α-glucosidase inhibitor, 0.0%; renin-angiotensin system blocker, 35.7%; diuretic, 66.7%; calcium channel blocker, 57.1%; statin, 38.1%; ezetimibe, 2.4%; potassium binder, 4.8%; phosphate binder, 19.0%; vitamin D analog, 16.7%; and calcimimetic, 2.4% (Table 1). The administered dose of elobixibat was 5 mg/day in 6 patients, 10 mg/day in 29 patients, and 15 mg/day in 7 patients.

**Table 1:** Baseline patient features.

		**Total patients (*n* = 42)**
Age (years)		72.5 ± 13.6
Male sex (number, %)		30 (71.4)
Body weight (kg)		59.5 ± 11.2
Body mass index (kg/m^2^)		23.0 ± 3.7
Systolic blood pressure (mmHg)		135.6 ± 14.8
Diastolic blood pressure (mmHg)		72.1 ± 15.3
CKD stage	G3 (number, %)	6 (14.3)
	G4 (number, %)	9 (21.4)
	G5 (number, %)	9 (21.4)
	G5D (number, %)	18 (42.9)
Other laxative use (number, %)		20 (47.6)
Concomitant laxative	Sennoside (number, %)	8 (19.0)
	Picosulfate (number, %)	5 (11.9)
	Magnesium oxide (number, %)	1 (2.4)
	Macrogol (number, %)	4 (9.5)
	Lubiprostone (number, %)	3 (7.1)
	Linaclotide (number, %)	4 (9.5)
Diabetes mellitus (number, %)		18 (42.9)
Dipeptidyl peptidase-4 inhibitor (number, %)		9 (21.4)
Glucagon-like peptide-1 receptor agonist (number, %)		0 (0.0)
Insulin (number, %)		6 (14.3)
Metformin (number, %)		0 (0.0)
α-Glucosidase inhibitor (number, %)		0 (0.0)
Renin-angiotensin system blocker (number, %)		15 (35.7)
Diuretic (number, %)		28 (66.7)
Calcium channel blocker (number, %)		24 (57.1)
Statin (number, %)		16 (38.1)
Ezetimibe (number, %)		1 (2.4)
Potassium binder (number, %)		2 (4.8)
Phosphate binder (number, %)		8 (19.0)
Vitamin D analog (number, %)		7 (16.7)
Calcimimetic (number, %)		1 (2.4)

*CKD, chronic kidney disease. Data are presented as mean ± standard deviation or number (%)*.

### Effect of Elobixibat on Constipation

Stool frequency significantly improved from 0.5 ± 0.4 per day before initiation of elobixibat to 1.1 ± 0.6 per day after initiation (*p* < 0.001) (Table 2). Improvement of stool frequency was similar between subgroups according to CKD stage (G3–G5 vs. G5D), concomitant laxative use (with vs. without), and timing of administration (before vs. after breakfast) (Table 2).

**Table 2:** Stool frequency (times per day) before and after 1 week of elobixibat administration according to CKD stage, concomitant laxative use, and timing of administration.

		**Before**	**After**	***p*-value**
	Total (*n* = 42)	0.5 ± 0.4	1.1 ± 0.6	<0.001
CKD stage	G3b–G5 (*n* = 24)	0.4 ± 0.3	1.1 ± 0.7	<0.001
	G5D (*n* = 18)	0.6 ± 0.4	1.0 ± 0.5	0.004
Other laxative use	With (*n* = 20)	0.5 ± 0.4	1.2 ± 0.7	0.002
	Without (*n* = 22)	0.4 ± 0.3	0.9 ± 0.5	<0.001
Timing of administration	After breakfast (*n* = 21)	0.4 ± 0.3	1.1 ± 0.7	<0.001
	Before breakfast (*n* = 21)	0.6 ± 0.4	1.0 ± 0.5	0.006

*CKD, chronic kidney disease. Data are presented as mean ± standard deviation*.

### Effect of Elobixibat on Lipid Metabolism

LDL-cholesterol concentration decreased significantly from 90.9 ± 37.2 mg/dL at baseline to 77.5 ± 34.8 mg/dL after 4 weeks of elobixibat administration (*p* < 0.05) (Table 3). In contrast, HDL-cholesterol concentration increased significantly from 44.9 ± 14.3 mg/dL at baseline to 57.0 ± 25.8 mg/dL after 4 weeks of elobixibat administration (*p* < 0.05) (Table 3). Total cholesterol and triglyceride concentrations did not change significantly after 4 weeks of elobixibat administration (Table 3).

**Table 3:** Clinical parameters before and after 4 weeks of elobixibat administration.

	**Baseline (*n* = 42)**	**After 4 weeks (*n* = 42)**	***p*-value**
Body weight (kg)	59.5 ± 11.2	57.4 ± 11.2	0.09
Albumin (g/dL)	3.2 ± 0.8	3.4 ± 0.7	0.021^*^
Hemoglobin (g/dL)	10.1 ± 1.7	10.4 ± 1.6	0.08
Alanine aminotransferase (IU/L)	10 (7–18)	14 (8–31)	0.33
Blood urea nitrogen (mg/dL)	54.9 ± 33.5	48.9 ± 20.8	0.62
Creatinine (mg/dL)	5.2 ± 3.6	4.7 ± 3.3	0.07
Sodium (mEq/L)	138.0 ± 3.5	137.2 ± 4.0	0.34
Potassium (mEq/L)	4.2 ± 0.6	4.0 ± 0.6	0.10
Chloride (mEq/L)	102.8 ± 4.8	102.8 ± 4.9	0.97
Total calcium (mg/dL)	8.4 ± 1.0	8.3 ± 0.9	0.64
Phosphate (mg/dL)	4.4 ± 1.5	3.9 ± 0.8	0.23
Magnesium (mg/dL)	2.1 ± 0.4	2.1 ± 0.4	0.85
Total cholesterol (mg/dL)	163.1 ± 44.2	162.0 ± 51.0	0.65
LDL-cholesterol (mg/dL)	90.9 ± 37.2	77.5 ± 34.8	0.044^*^
HDL-cholesterol (mg/dL)	44.9 ± 14.3	57.0 ± 25.8	0.037^*^
Triglyceride (mg/dL)	129.1 ± 46.6	116.0 ± 42.7	0.39
Uric acid (mg/dL)	6.6 ± 2.4	6.5 ± 2.0	0.86
HbA1c (%)	6.2 ± 1.2	6.2 ± 1.1	0.68

*HbA1c, serum hemoglobin A1c; HDL, high-density lipoprotein; LDL, low-density lipoprotein. Valuables are presented as mean ± standard deviation or median [interquartile range]*.

* *P-values are statistically significant*.

### Factors Associated With the Change in Stool Frequency After Initiation of Elobixibat Administration

Simple linear regression analysis revealed that the change in stool frequency after initiation of elobixibat significantly correlated with phosphate concentration (standard coefficient = 0.321, *p* = 0.043) (Table 4).

**Table 4:** Simple linear regression analyses of the variables correlated with the change in stool frequency after initiation of elobixibat administration.

**Variables**	**Simple linear regression analysis**	
	**Standard coefficient**	***p*-value**
Age (years)	0.009	0.95
Male sex (yes vs. no)	−0.143	0.36
Body weight (kg)	−0.179	0.26
Body mass index (kg/m^2^)	−0.114	0.47
Systolic blood pressure (mmHg)	−0.208	0.19
Diastolic blood pressure (mmHg)	−0.077	0.63
CKD stage (G3b–G5 vs. G5D)	−0.284	0.07
Other laxative use (yes vs. no)	0.126	0.43
Timing of administration (after breakfast vs. before breakfast)	0.260	0.10
Diabetes mellitus (yes vs. no)	−0.107	0.50
Dipeptidyl peptidase-4 inhibitor (yes vs. no)	−0.055	0.73
Glucagon-like peptide-1 receptor agonist (yes vs. no)	0.000	—
Insulin (yes vs. no)	−0.148	0.35
Renin-angiotensin system blocker (yes vs. no)	−0.209	0.18
Diuretic (yes vs. no)	−0.142	0.37
Calcium channel blocker (yes vs. no)	−0.034	0.83
Statin (yes vs. no)	−0.106	0.50
Ezetimibe (yes vs. no)	−0.065	0.68
Potassium binder (yes vs. no)	−0.230	0.14
Phosphate binder (yes vs. no)	−0.201	0.20
Vitamin D analog (yes vs. no)	−0.195	0.22
Calcimimetic (yes vs. no)	−0.281	0.07
Albumin (g/dL)	0.132	0.41
Hemoglobin (g/dL)	0.233	0.14
Alanine aminotransferase (IU/L)	0.039	0.81
Blood urea nitrogen (mg/dL)	0.239	0.13
Creatinine (mg/dL)	−0.148	0.36
Sodium (mEq/L)	0.190	0.24
Potassium (mEq/L)	0.094	0.56
Chloride (mEq/L)	−0.030	0.85
Total calcium (mg/dL)	0.052	0.75
Phosphate (mg/dL)	0.321	0.043^*^
Magnesium (mg/dL)	0.168	0.31
Total cholesterol (mg/dL)	0.226	0.17
LDL-cholesterol (mg/dL)	0.195	0.25
HDL-cholesterol (mg/dL)	0.298	0.07
Triglyceride (mg/dL)	−0.105	0.54
Uric acid (mg/dL)	0.109	0.50
HbA1c (%)	0.232	0.19

*CKD, chronic kidney disease; HbA1c, serum hemoglobin A1c; HDL, high-density lipoprotein; LDL, low-density lipoprotein*.

* *P-value is statistically significant*.

### Changes in Other Clinical Parameters and Adverse Effects

There were no changes in body weight, hemoglobin, serum alanine aminotransferase activity, serum creatinine, sodium, potassium, chloride, total calcium, phosphate, magnesium, serum uric acid, or HbA1c concentration between baseline and after 4 weeks of elobixibat administration (Table 3). Serum albumin concentration increased significantly from 3.2 ± 0.8 g/dL to 3.4 ± 0.7 g/dL after 4 weeks of elobixibat administration (*p* < 0.05) (Table 3). Adverse effects were observed in two participants: nausea in one and diarrhea in another. However, they were tolerant of elobixibat and its administration was continued.

## Discussion

In the present study, we found that elobixibat improved constipation and lipid metabolism in patients with renal impairment, without serious adverse events. We also found that phosphate concentration was correlated with the change in stool frequency after initiation of elobixibat.

A recent phase III clinical study showed that elobixibat increased the frequency of spontaneous bowel movements in non-renal impaired patients with chronic constipation (9). An observational study reported that elobixibat increased the number of spontaneous bowel movements in patients with chronic constipation undergoing hemodialysis (13). In the present study, elobixibat increased stool frequency in patients with moderate to end-stage CKD, and its effect on improving constipation was similar between patients with CKD stages G3–G5 and G5D. These results suggest that elobixibat improves constipation in both patients with and without renal impairment. In the present study, the change in stool frequency after initiation of elobixibat was positively correlated with phosphate concentration. Recently, bile acids were suggested to contribute to intestinal phosphate absorption in a vitamin D receptor-dependent manner (14). Elobixibat inhibits the uptake of bile acids in the terminal ileum and increases the amount of bile acid in the intestine lumen (8). Increased bile acids can enhance bowel movement and phosphate absorption. This might explain the positive association between the change in stool frequency after initiation of elobixibat and phosphate concentration in this study. Further studies are needed to clarify the effect of elobixibat on phosphate metabolism in patients with renal impairment.

Elobixibat is minimally absorbed and acts locally in the lumen of the gastrointestinal tract. It binds and inhibits bile acid transporters in the ileal mucosa, thereby increasing bile acid influx into the colon (8). Bile acids are excreted from the liver as conjugates, stored in the gall bladder, and released into the duodenum by postprandial contraction of the gallbladder (15). Therefore, for maximum effect, elobixibat administration is recommended before breakfast (10). However, in the present study, the effect of elobixibat on constipation did not differ between administration before or after breakfast. In a real-world clinical setting, elobixibat may improve constipation in patients with renal impairment regardless of whether it is administered before or after breakfast. Further studies are necessary to investigate whether the effect of elobixibat is similar between its administration before or after breakfast.

Elobixibat is often used concomitantly with other laxatives in patients with chronic constipation (16). A retrospective observational study reported that the proportion of other laxatives used was not different between responders and non-responders to elobixibat in a cohort of older patients with chronic constipation (16). In the present study, the effect of elobixibat on constipation did not differ between patients with and without concomitant laxative use. Elobixibat may improve constipation in patients with renal impairment regardless of whether they use other laxatives. Further studies are necessary to determine whether elobixibat is similarly effective between patients with chronic constipation with and without concomitant laxative use.

Elobixibat increases excretion of bile acids into feces by inhibiting ileal bile acid transporters, which enhances production of bile acids from cholesterol in the liver (15). This subsequently leads to reduction in serum cholesterol concentration (15). It was reported that elobixibat reduced LDL-cholesterol concentration in patients without renal impairment (10). In the present study, elobixibat decreased LDL-cholesterol concentration and increased HDL-cholesterol concentration in patients with renal impairment. It has been suggested that the reduction of triglyceride-rich lipoproteins by the reduction in cholesterol delivery from the intestine to the liver leads to a decreased rate of cholesterol transfer from HDL particles, thereby increasing HDL-cholesterol concentration (17). These results suggest elobixibat may improve lipid metabolism in both patients with and without renal impairment. Further studies are necessary to investigate the effect of elobixibat on lipid metabolism in patients with renal impairment.

Elobixibat reportedly increases water excretion in feces through increased bile acid excretion by inhibiting bile acid transporters (8). In the present study, serum albumin concentration was increased after 4 weeks of elobixibat administration. Hemoglobin concentration also increased and body weight decreased, although not significantly. However, renal function did not change after administration of elobixibat. It was reported that elobixibat reduces interdialytic body weight gain in patients undergoing hemodialysis (18). These findings suggest elobixibat reduces excess water by excreting it into feces. Further studies are necessary to investigate the effect of elobixibat on body fluid volume in patients with renal impairment.

The present study had several limitations. First, this was a retrospective observational study. Therefore, selection bias could not be fully eliminated. Second, the study was performed at a single center and the number of participants was small, which limits the external validity of the results. Third, this was a before-after study without a control group, and the observation period was short. Fourth, we could not assess stool consistency in this study because of the retrospective nature, although stool consistency was important as well as stool frequency in this kind of study (9, 10). Fifth, there might have been the confounding effect of concomitant laxatives, although the patients' use of concomitant laxatives did not change during the study period. Sixth, very few patients are included in the different subgroups of the study. Therefore, the results are interpreted as a pilot study and cannot be extrapolated to the group of CKD patients, until studies with a larger sample number are carried out. Therefore, large-scale, long-term, multicenter randomized clinical studies are required to confirm the efficacy of elobixibat for improvement of constipation and lipid metabolism in patients with renal impairment.

In conclusion, elobixibat improved constipation and lipid metabolism in patients with moderate to end-stage CKD, without serious adverse events. The effect of elobixibat on improving constipation was similar in patients, regardless of whether they were undergoing dialysis, on concomitant laxatives, or were administered elobixibat before or after breakfast.

## Data Availability Statement

The raw data supporting the conclusions of this article will be made available by the authors, without undue reservation.

## Ethics Statement

The studies involving human participants were reviewed and approved by Ethics Committee of Saitama Medical Center, Jichi Medical University. Written informed consent for participation was not required for this study in accordance with the national legislation and the institutional requirements.

## Author Contributions

KH and HM conceived and designed the study. HN, MU, JM, SK, SM, YMu, TK, and AA collected the data. KY and HI performed the statistical analysis. MM wrote the first draft of the manuscript. KI, YU, and SO made critical revisions. YMo approved the final version. All authors contributed to this manuscript and approved the final version for submission.

## Conflict of Interest

The authors declare that the research was conducted in the absence of any commercial or financial relationships that could be construed as a potential conflict of interest.

## Publisher's Note

All claims expressed in this article are solely those of the authors and do not necessarily represent those of their affiliated organizations, or those of the publisher, the editors and the reviewers. Any product that may be evaluated in this article, or claim that may be made by its manufacturer, is not guaranteed or endorsed by the publisher.

## References

[1] Sumida K Molnar MZ Potukuchi PK Thomas F Lu JL Matsushita K. Constipation and incident CKD. J Am Soc Nephrol. (2017) 28:1248–58. 10.1681/ASN.2016060656 28122944 PMC5373459

[2] Sumida K Molnar MZ Potukuchi PK Thomas F Lu JL Yamagata K. Constipation and risk of death and cardiovascular events. Atherosclerosis. (2019) 281:114–20. 10.1016/j.atherosclerosis.2018.12.021 30658186 PMC6399019

[3] Belsey J Greenfield S Candy D Geraint M. Systematic review: impact of constipation on quality of life in adults and children. Aliment Pharmacol Ther. (2010) 31:938–49. 10.1111/j.1365-2036.2010.04273.x 20180788

[4] Lim J Park H Lee H Lee E Lee D Jung HW. Higher frailty burden in older adults with chronic constipation. BMC Gastroenterol. (2021) 21:137. 10.1186/s12876-021-01684-x 33765938 PMC7995705

[5] Mihara H Murayama A Nanjo S Ando T Tajiri K Fujinami H. Factors correlated with drug use for constipation: perspectives from the 2016 open Japanese National Database. BMC Gastroenterol. (2020) 20:284. 10.1186/s12876-020-01425-6 32831027 PMC7444268

[6] Mori H Tack J Suzuki H. Magnesium oxide in constipation. Nutrients. (2021) 13:421. 10.3390/nu13020421 33525523 PMC7911806

[7] Xing JH Soffer EE. Adverse effects of laxatives. Dis Colon Rectum. (2001) 44:1201–9. 10.1007/BF02234645 11535863

[8] Chedid V Vijayvargiya P Camilleri M. Elobixibat for the treatment of constipation. Expert Rev Gastroenterol Hepatol. (2018) 12:951–60. 10.1080/17474124.2018.1522248 30204504 PMC6386599

[9] Nakajima A Seki M Taniguchi S Ohta A Gillberg PG Mattsson JP. Safety and efficacy of elobixibat for chronic constipation: results from a randomised, double-blind, placebo-controlled, phase 3 trial and an open-label, single-arm, phase 3 trial. Lancet Gastroenterol Hepatol. (2018) 3:537–47. 10.1016/S2468-1253(18)30123-7 29805116

[10] Kumagai Y Amano H Sasaki Y Nakagawa C Maeda M Oikawa I. Effect of single and multiple doses of elobixibat, an ileal bile acid transporter inhibitor, on chronic constipation: a randomized controlled trial. Br J Clin Pharmacol. (2018) 84:2393–404. 10.1111/bcp.13698 29959787 PMC6138487

[11] Mearin F Lacy BE Chang L Chey WD Lembo AJ Simren M. Bowel disorders. Gastroenterology. (2016) 150:1393–407. 10.1053/j.gastro.2016.02.031 27144627

[12] Zhao YF Guo XJ Zhang ZS Ma XQ Wang R Yan XY. Epidemiology of functional diarrhea and comparison with diarrhea-predominant irritable bowel syndrome: a population-based survey in China. PLoS ONE. (2012) 7:e43749. 10.1371/journal.pone.0043749 22937091 PMC3427143

[13] Kamei D Kamei Y Nagano M Mineshima M Nitta K Tsuchiya K. Elobixibat alleviates chronic constipation in hemodialysis patients: a questionnaire-based study. BMC Gastroenterol. (2020) 20:26. 10.1186/s12876-020-1179-6 32005162 PMC6995167

[14] Hashimoto N Matsui I Ishizuka S Inoue K Matsumoto A Shimada K. Lithocholic acid increases intestinal phosphate and calcium absorption in a vitamin D receptor dependent but transcellular pathway independent manner. Kidney Int. (2020) 97:1164–80. 10.1016/j.kint.2020.01.032 32354638

[15] Acosta A Camilleri M. Elobixibat and its potential role in chronic idiopathic constipation. Therap Adv Gastroenterol. (2014) 7:167–75. 10.1177/1756283X14528269 25057297 PMC4107709

[16] Tomie A Yoshida N Kugai M Hirose R Dohi O Inoue K. The efficacy and safety of elobixibat for the elderly with chronic constipation: a multicenter retrospective cohort study. Gastroenterol Res Pract. (2020) 2020:9656040. 10.1155/2020/9656040 32411210 PMC7204088

[17] Bays HE Neff D Tomassini JE Tershakovec AM. Ezetimibe: cholesterol lowering and beyond. Expert Rev Cardiovasc Ther. (2008) 6:447–70. 10.1586/14779072.6.4.447 18402536

[18] Shono T Hyakutake H. Efficacy and safety of elobixibat in hemodialysis patients with chronic constipation: a retrospective study. Renal Replacement Therapy. (2020) 6:21. 10.1186/s41100-020-00267-y

